# Supplementary material for: Identification of a novel mycovirus belonging to the “flexivirus”-related family with icosahedral virion
Source: Virus Evol. 2024 Nov 6;10(1):veae093. doi: 10.1093/ve/veae093 (PMC11654247; doi:10.1093/ve/veae093)
Supplement: veae093_Supp [file veae093_supp.zip › FoIV1_FigureS2.pptx]

## Slide 1
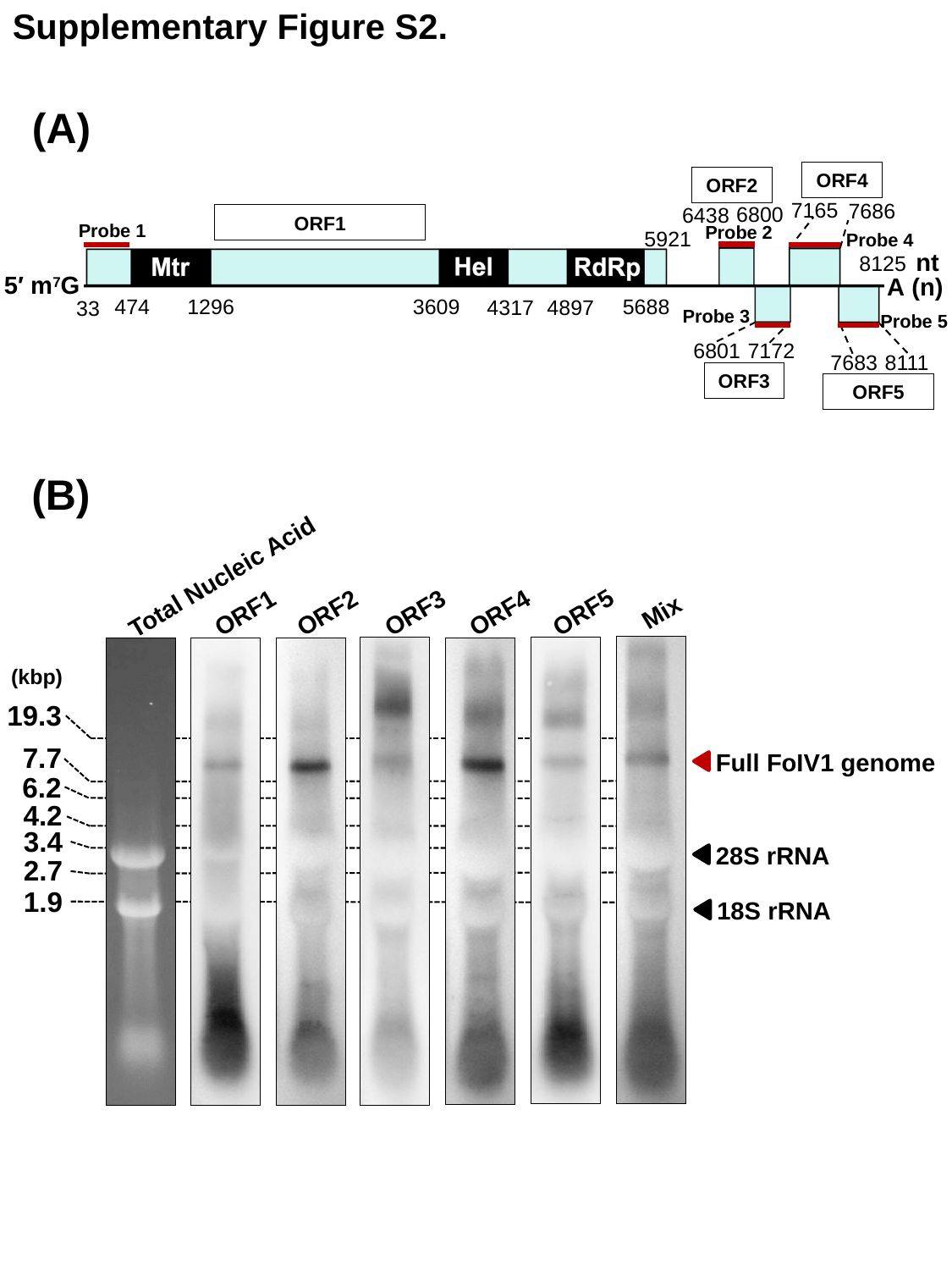

Supplementary Figure S2.
(A)
ORF4
ORF2
7165
7686
6800
6438
ORF1
Probe 1
Probe 2
5921
Probe 4
nt
8125
5′ m7G
A (n)
474
1296
3609
5688
4897
4317
33
Probe 3
Probe 5
7172
6801
8111
7683
ORF3
ORF5
(B)
Total Nucleic Acid
ORF5
Mix
ORF1
ORF2
ORF4
ORF3
(kbp)
19.3
7.7
Full FoIV1 genome
6.2
4.2
3.4
28S rRNA
2.7
1.9
18S rRNA

## Slide 2
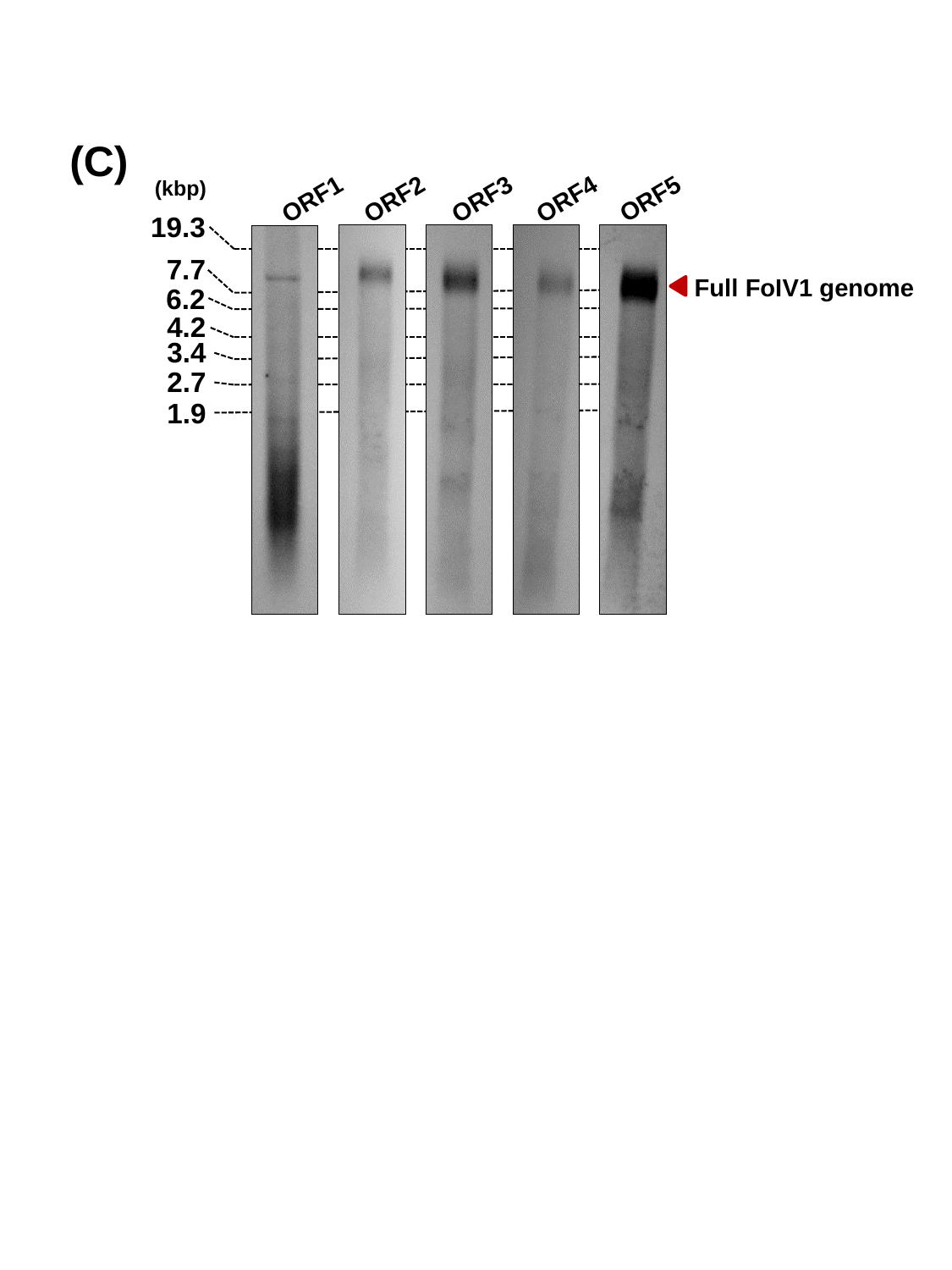

(C)
(kbp)
ORF5
ORF1
ORF2
ORF4
ORF3
19.3
7.7
Full FoIV1 genome
6.2
4.2
3.4
2.7
1.9
