# Supplementary material for: Identification of a novel mycovirus belonging to the “flexivirus”-related family with icosahedral virion
Source: Virus Evol. 2024 Nov 6;10(1):veae093. doi: 10.1093/ve/veae093 (PMC11654247; doi:10.1093/ve/veae093)
Supplement: veae093_Supp [file veae093_supp.zip › FoIV1_FigureS3.pptx]

## Slide 1
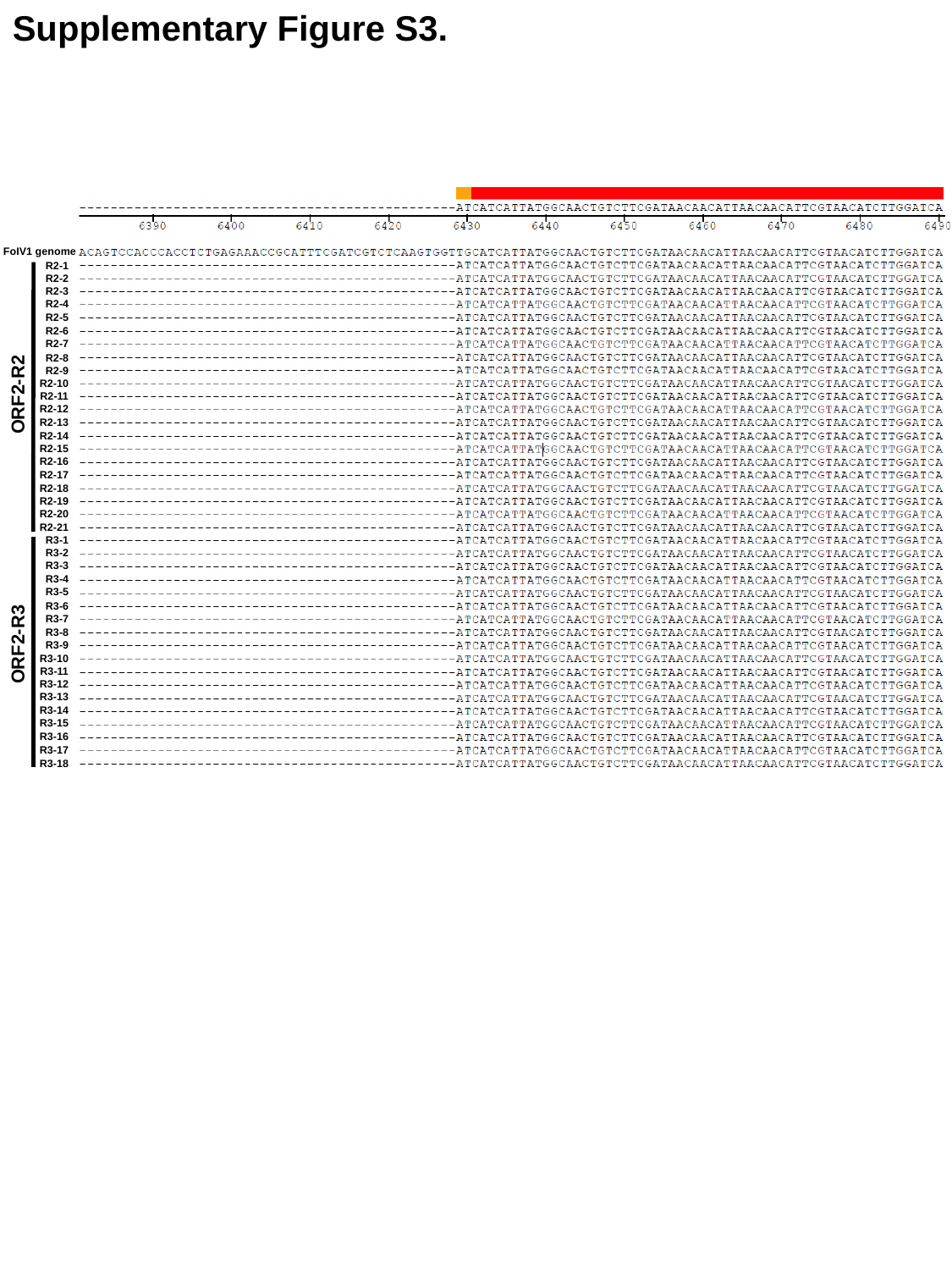

Supplementary Figure S3.
FoIV1 genome
R2-1
R2-2
R2-3
R2-4
R2-5
R2-6
R2-7
R2-8
R2-9
R2-10
ORF2-R2
R2-11
R2-12
R2-13
R2-14
R2-15
R2-16
R2-17
R2-18
R2-19
R2-20
R2-21
R3-1
R3-2
R3-3
R3-4
R3-5
R3-6
R3-7
R3-8
ORF2-R3
R3-9
R3-10
R3-11
R3-12
R3-13
R3-14
R3-15
R3-16
R3-17
R3-18
