# Supplementary material for: Identification of a novel mycovirus belonging to the “flexivirus”-related family with icosahedral virion
Source: Virus Evol. 2024 Nov 6;10(1):veae093. doi: 10.1093/ve/veae093 (PMC11654247; doi:10.1093/ve/veae093)
Supplement: veae093_Supp [file veae093_supp.zip › FoIV1_FigureS5.pptx]

## Slide 1
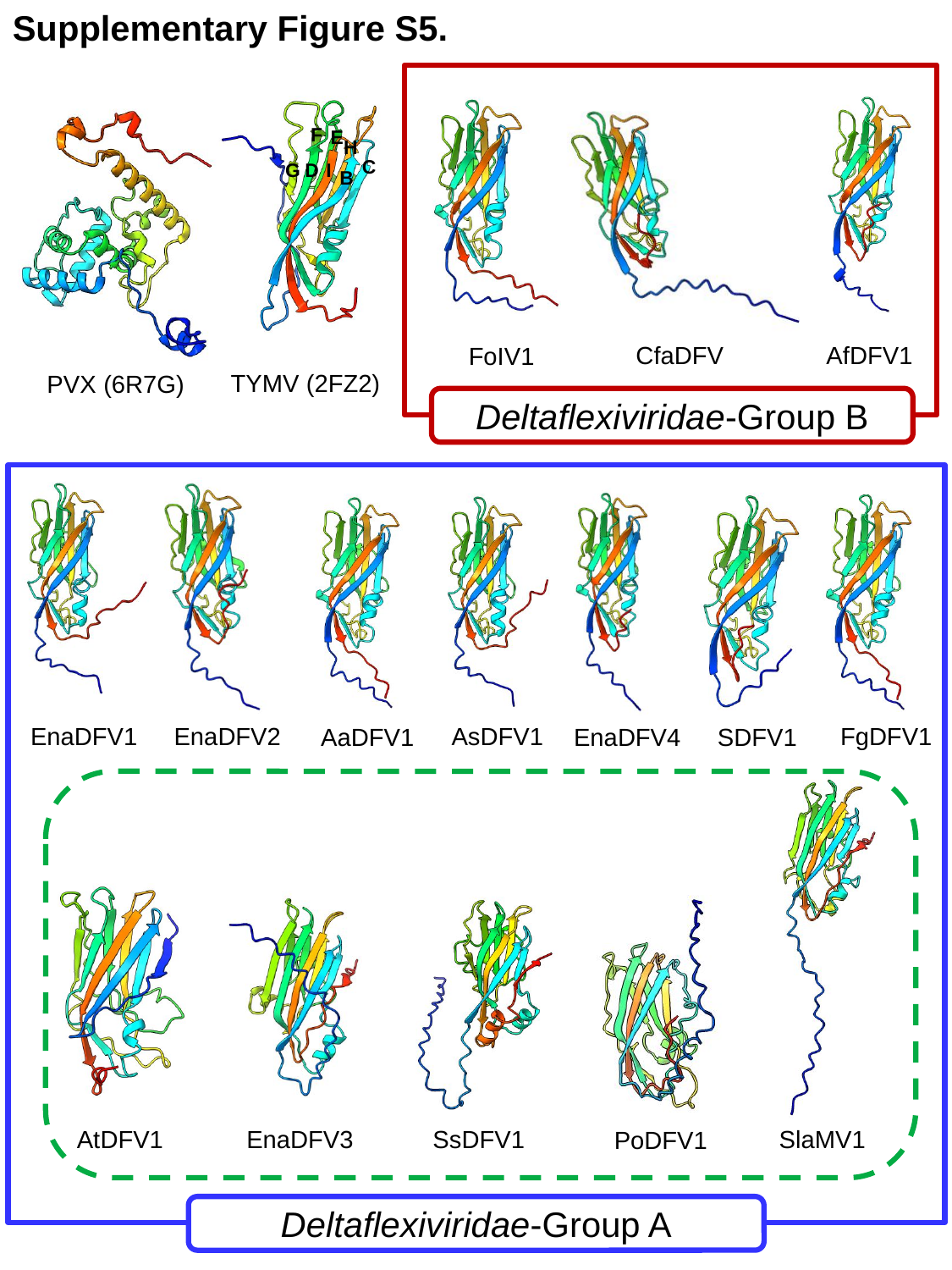

Supplementary Figure S5.
F
E
H
C
G
D
I
B
AfDFV1
CfaDFV
FoIV1
TYMV (2FZ2)
PVX (6R7G)
Deltaflexiviridae-Group B
EnaDFV1
FgDFV1
EnaDFV2
AsDFV1
SDFV1
EnaDFV4
AaDFV1
AtDFV1
EnaDFV3
SsDFV1
SlaMV1
PoDFV1
Deltaflexiviridae-Group A
