# Supplementary material for: Identification of a novel mycovirus belonging to the “flexivirus”-related family with icosahedral virion
Source: Virus Evol. 2024 Nov 6;10(1):veae093. doi: 10.1093/ve/veae093 (PMC11654247; doi:10.1093/ve/veae093)
Supplement: veae093_Supp [file veae093_supp.zip › FoIV1_TableS1_revision.pptx]

## Slide 1
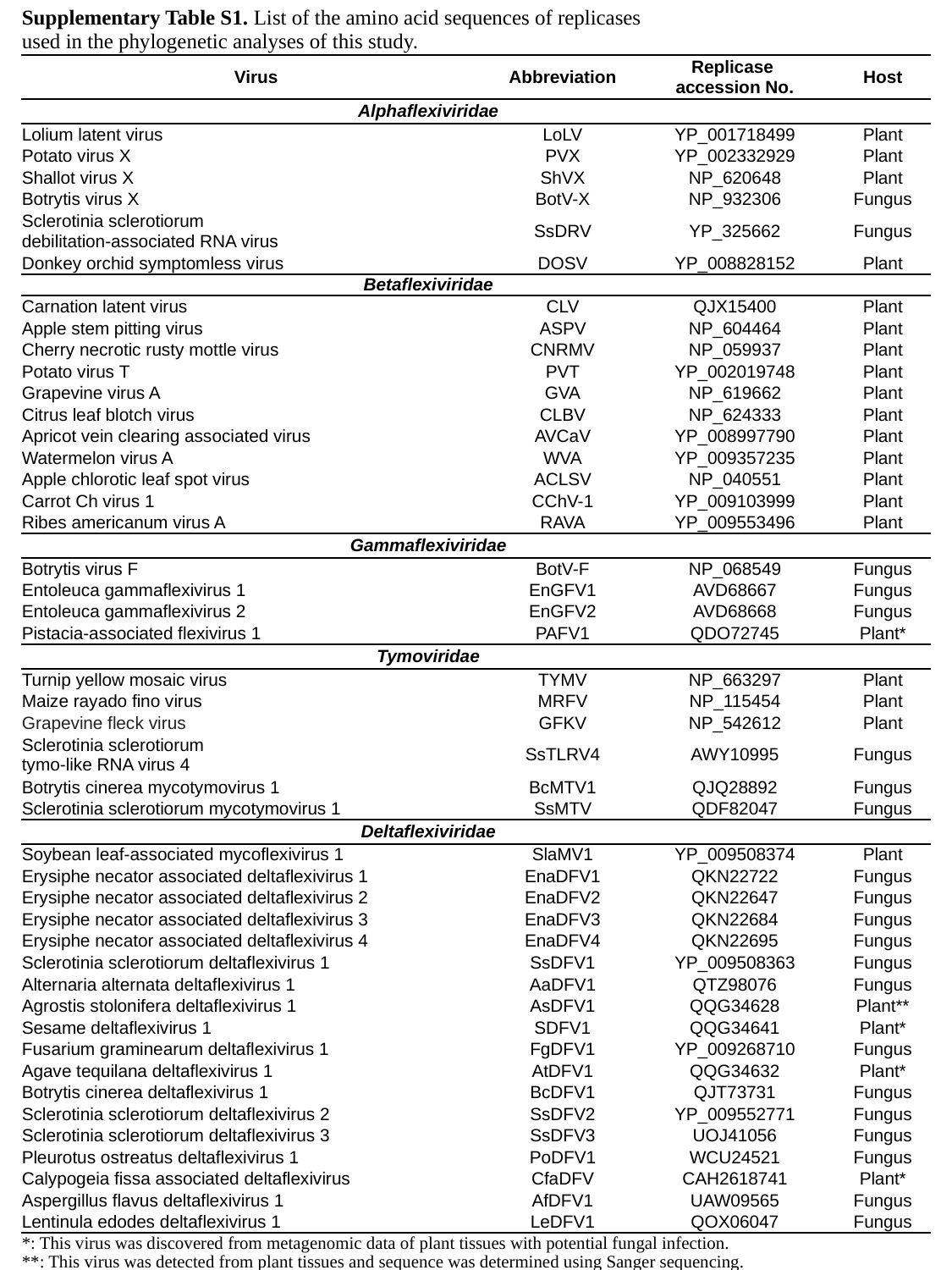

| Supplementary Table S1. List of the amino acid sequences of replicases used in the phylogenetic analyses of this study. | | | |
| --- | --- | --- | --- |
| Virus | Abbreviation | Replicase accession No. | Host |
| Alphaflexiviridae | | | |
| Lolium latent virus | LoLV | YP\_001718499 | Plant |
| Potato virus X | PVX | YP\_002332929 | Plant |
| Shallot virus X | ShVX | NP\_620648 | Plant |
| Botrytis virus X | BotV-X | NP\_932306 | Fungus |
| Sclerotinia sclerotiorum debilitation-associated RNA virus | SsDRV | YP\_325662 | Fungus |
| Donkey orchid symptomless virus | DOSV | YP\_008828152 | Plant |
| Betaflexiviridae | | | |
| Carnation latent virus | CLV | QJX15400 | Plant |
| Apple stem pitting virus | ASPV | NP\_604464 | Plant |
| Cherry necrotic rusty mottle virus | CNRMV | NP\_059937 | Plant |
| Potato virus T | PVT | YP\_002019748 | Plant |
| Grapevine virus A | GVA | NP\_619662 | Plant |
| Citrus leaf blotch virus | CLBV | NP\_624333 | Plant |
| Apricot vein clearing associated virus | AVCaV | YP\_008997790 | Plant |
| Watermelon virus A | WVA | YP\_009357235 | Plant |
| Apple chlorotic leaf spot virus | ACLSV | NP\_040551 | Plant |
| Carrot Ch virus 1 | CChV-1 | YP\_009103999 | Plant |
| Ribes americanum virus A | RAVA | YP\_009553496 | Plant |
| Gammaflexiviridae | | | |
| Botrytis virus F | BotV-F | NP\_068549 | Fungus |
| Entoleuca gammaflexivirus 1 | EnGFV1 | AVD68667 | Fungus |
| Entoleuca gammaflexivirus 2 | EnGFV2 | AVD68668 | Fungus |
| Pistacia-associated flexivirus 1 | PAFV1 | QDO72745 | Plant\* |
| Tymoviridae | | | |
| Turnip yellow mosaic virus | TYMV | NP\_663297 | Plant |
| Maize rayado fino virus | MRFV | NP\_115454 | Plant |
| Grapevine fleck virus | GFKV | NP\_542612 | Plant |
| Sclerotinia sclerotiorum tymo-like RNA virus 4 | SsTLRV4 | AWY10995 | Fungus |
| Botrytis cinerea mycotymovirus 1 | BcMTV1 | QJQ28892 | Fungus |
| Sclerotinia sclerotiorum mycotymovirus 1 | SsMTV | QDF82047 | Fungus |
| Deltaflexiviridae | | | |
| Soybean leaf-associated mycoflexivirus 1 | SlaMV1 | YP\_009508374 | Plant |
| Erysiphe necator associated deltaflexivirus 1 | EnaDFV1 | QKN22722 | Fungus |
| Erysiphe necator associated deltaflexivirus 2 | EnaDFV2 | QKN22647 | Fungus |
| Erysiphe necator associated deltaflexivirus 3 | EnaDFV3 | QKN22684 | Fungus |
| Erysiphe necator associated deltaflexivirus 4 | EnaDFV4 | QKN22695 | Fungus |
| Sclerotinia sclerotiorum deltaflexivirus 1 | SsDFV1 | YP\_009508363 | Fungus |
| Alternaria alternata deltaflexivirus 1 | AaDFV1 | QTZ98076 | Fungus |
| Agrostis stolonifera deltaflexivirus 1 | AsDFV1 | QQG34628 | Plant\*\* |
| Sesame deltaflexivirus 1 | SDFV1 | QQG34641 | Plant\* |
| Fusarium graminearum deltaflexivirus 1 | FgDFV1 | YP\_009268710 | Fungus |
| Agave tequilana deltaflexivirus 1 | AtDFV1 | QQG34632 | Plant\* |
| Botrytis cinerea deltaflexivirus 1 | BcDFV1 | QJT73731 | Fungus |
| Sclerotinia sclerotiorum deltaflexivirus 2 | SsDFV2 | YP\_009552771 | Fungus |
| Sclerotinia sclerotiorum deltaflexivirus 3 | SsDFV3 | UOJ41056 | Fungus |
| Pleurotus ostreatus deltaflexivirus 1 | PoDFV1 | WCU24521 | Fungus |
| Calypogeia fissa associated deltaflexivirus | CfaDFV | CAH2618741 | Plant\* |
| Aspergillus flavus deltaflexivirus 1 | AfDFV1 | UAW09565 | Fungus |
| Lentinula edodes deltaflexivirus 1 | LeDFV1 | QOX06047 | Fungus |
| \*: This virus was discovered from metagenomic data of plant tissues with potential fungal infection. \*\*: This virus was detected from plant tissues and sequence was determined using Sanger sequencing. | | | |
