# Supplementary material for: Identification of a novel mycovirus belonging to the “flexivirus”-related family with icosahedral virion
Source: Virus Evol. 2024 Nov 6;10(1):veae093. doi: 10.1093/ve/veae093 (PMC11654247; doi:10.1093/ve/veae093)
Supplement: veae093_Supp [file veae093_supp.zip › FoIV1_TableS2.pptx]

## Slide 1
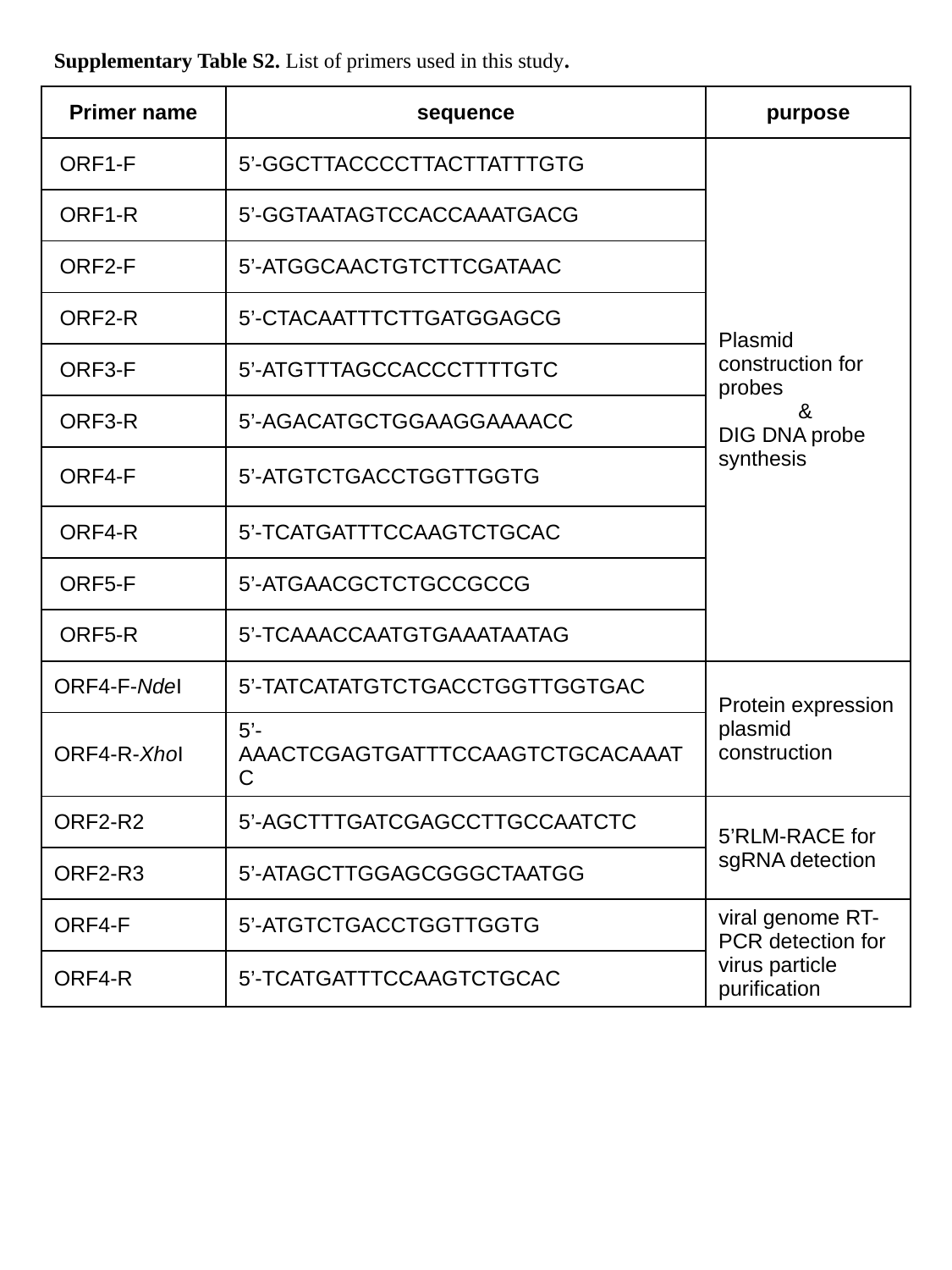

| Supplementary Table S2. List of primers used in this study. | | |
| --- | --- | --- |
| Primer name | sequence | purpose |
| ORF1-F | 5’-GGCTTACCCCTTACTTATTTGTG | Plasmid construction for probes & DIG DNA probe synthesis |
| ORF1-R | 5’-GGTAATAGTCCACCAAATGACG | |
| ORF2-F | 5’-ATGGCAACTGTCTTCGATAAC | |
| ORF2-R | 5’-CTACAATTTCTTGATGGAGCG | |
| ORF3-F | 5’-ATGTTTAGCCACCCTTTTGTC | |
| ORF3-R | 5’-AGACATGCTGGAAGGAAAACC | |
| ORF4-F | 5’-ATGTCTGACCTGGTTGGTG | |
| ORF4-R | 5’-TCATGATTTCCAAGTCTGCAC | |
| ORF5-F | 5’-ATGAACGCTCTGCCGCCG | |
| ORF5-R | 5’-TCAAACCAATGTGAAATAATAG | |
| ORF4-F-NdeI | 5’-TATCATATGTCTGACCTGGTTGGTGAC | Protein expression plasmid construction |
| ORF4-R-XhoI | 5’-AAACTCGAGTGATTTCCAAGTCTGCACAAATC | |
| ORF2-R2 | 5’-AGCTTTGATCGAGCCTTGCCAATCTC | 5’RLM-RACE for sgRNA detection |
| ORF2-R3 | 5’-ATAGCTTGGAGCGGGCTAATGG | |
| ORF4-F | 5’-ATGTCTGACCTGGTTGGTG | viral genome RT-PCR detection for virus particle purification |
| ORF4-R | 5’-TCATGATTTCCAAGTCTGCAC | |
