# Supplementary material for: Identification of a novel mycovirus belonging to the “flexivirus”-related family with icosahedral virion
Source: Virus Evol. 2024 Nov 6;10(1):veae093. doi: 10.1093/ve/veae093 (PMC11654247; doi:10.1093/ve/veae093)
Supplement: veae093_Supp [file veae093_supp.zip › FoIV1_TableS3_revision.pptx]

## Slide 1
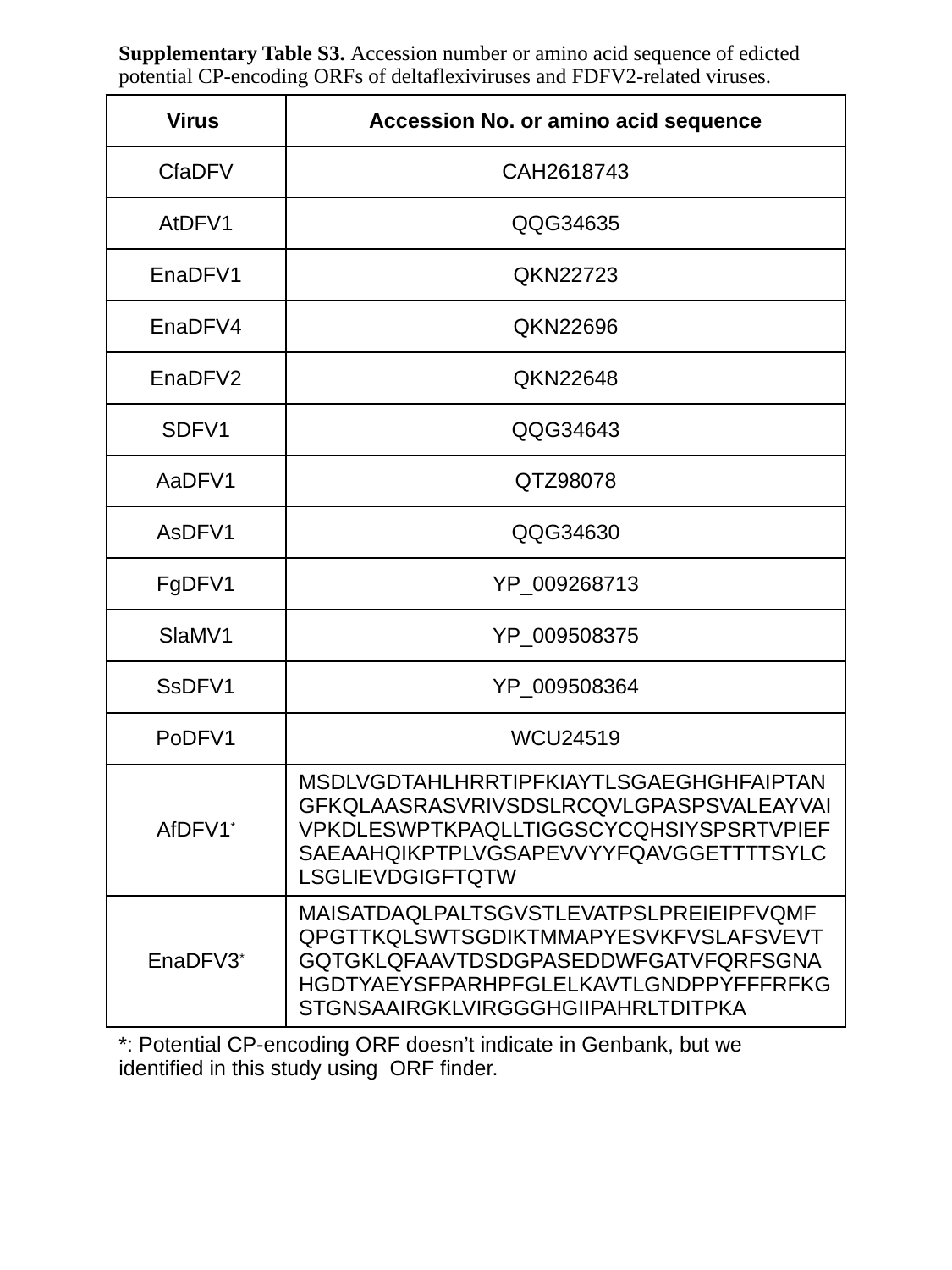

| Supplementary Table S3. Accession number or amino acid sequence of edicted potential CP-encoding ORFs of deltaflexiviruses and FDFV2-related viruses. | |
| --- | --- |
| Virus | Accession No. or amino acid sequence |
| CfaDFV | CAH2618743 |
| AtDFV1 | QQG34635 |
| EnaDFV1 | QKN22723 |
| EnaDFV4 | QKN22696 |
| EnaDFV2 | QKN22648 |
| SDFV1 | QQG34643 |
| AaDFV1 | QTZ98078 |
| AsDFV1 | QQG34630 |
| FgDFV1 | YP\_009268713 |
| SlaMV1 | YP\_009508375 |
| SsDFV1 | YP\_009508364 |
| PoDFV1 | WCU24519 |
| AfDFV1\* | MSDLVGDTAHLHRRTIPFKIAYTLSGAEGHGHFAIPTANGFKQLAASRASVRIVSDSLRCQVLGPASPSVALEAYVAIVPKDLESWPTKPAQLLTIGGSCYCQHSIYSPSRTVPIEFSAEAAHQIKPTPLVGSAPEVVYYFQAVGGETTTTSYLCLSGLIEVDGIGFTQTW |
| EnaDFV3\* | MAISATDAQLPALTSGVSTLEVATPSLPREIEIPFVQMFQPGTTKQLSWTSGDIKTMMAPYESVKFVSLAFSVEVTGQTGKLQFAAVTDSDGPASEDDWFGATVFQRFSGNAHGDTYAEYSFPARHPFGLELKAVTLGNDPPYFFFRFKGSTGNSAAIRGKLVIRGGGHGIIPAHRLTDITPKA |
| \*: Potential CP-encoding ORF doesn’t indicate in Genbank, but we identified in this study using ORF finder. | |
