# Supplementary figures and images for: Identification of a novel mycovirus belonging to the “flexivirus”-related family with icosahedral virion
Source: Virus Evol. 2024 Nov 6;10(1):veae093. doi: 10.1093/ve/veae093 (PMC11654247; doi:10.1093/ve/veae093)

## Slide 1
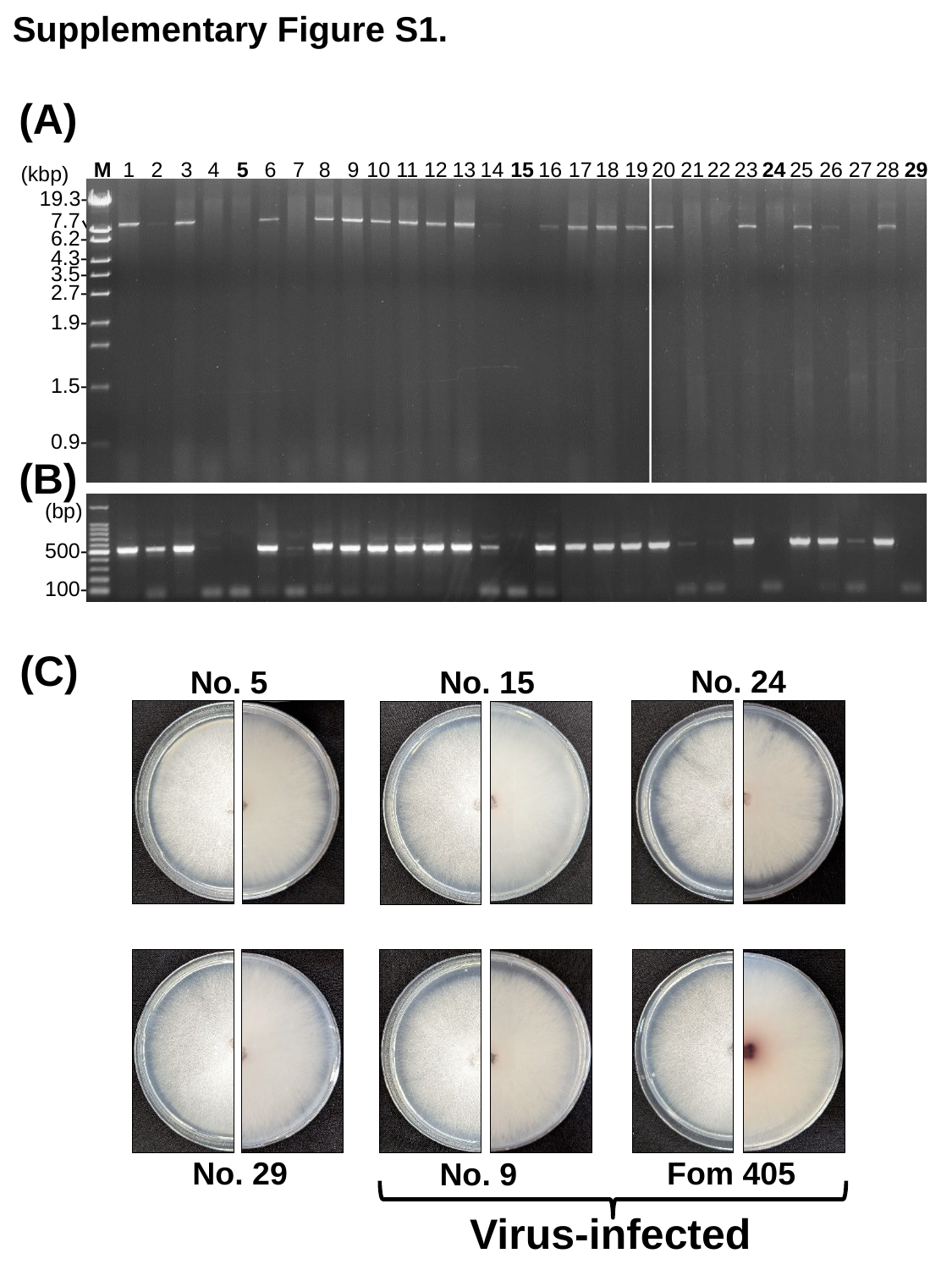

Supplementary Figure S1.
(A)
M
21
22
23
24
25
26
27
28
29
14
15
16
18
19
20
12
13
17
11
10
9
2
6
7
8
4
5
3
1
(kbp)
19.3-
7.7
6.2-
4.3-
3.5-
2.7-
1.9-
1.5-
0.9-
(B)
(bp)
500-
100-
(C)
No. 24
No. 15
No. 5
No. 29
Fom 405
No. 9
Virus-infected

Supplement: veae093_Supp [file veae093_supp.zip › FoIV1_FigureS1.pptx]

## Slide 1
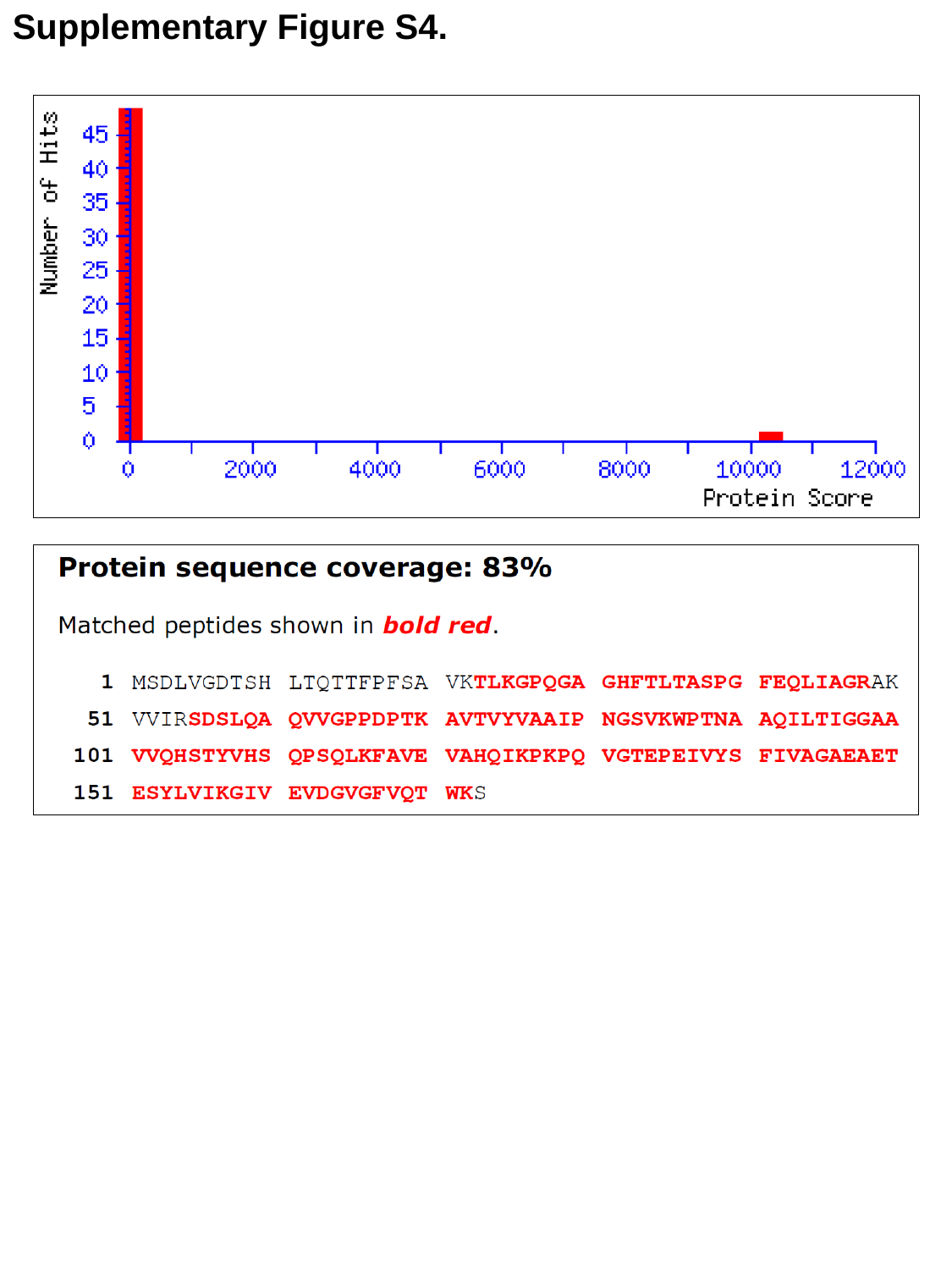

Supplementary Figure S4.

Supplement: veae093_Supp [file veae093_supp.zip › FoIV1_FigureS4.pptx]

## Slide 1
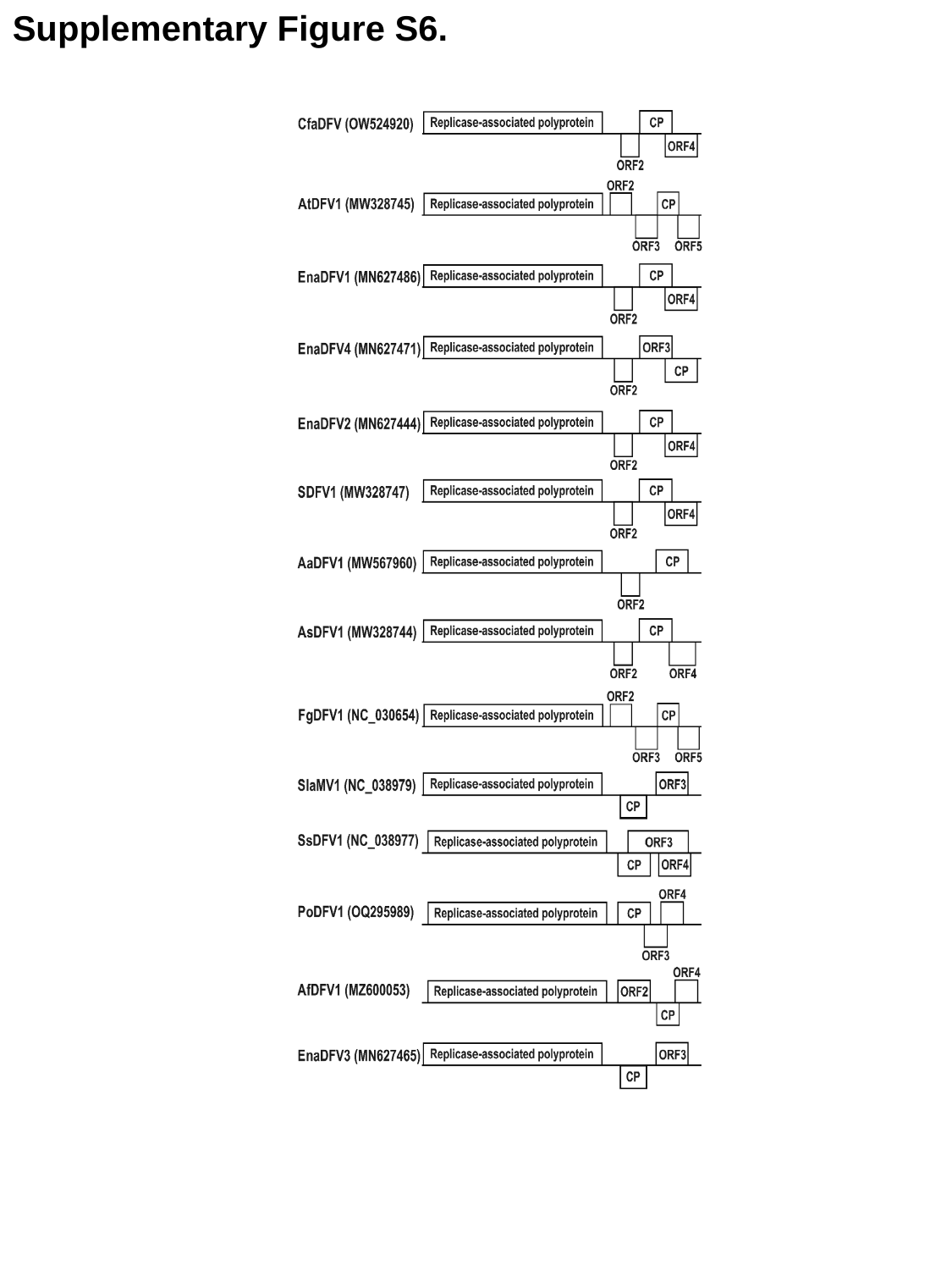

Supplementary Figure S6.

Supplement: veae093_Supp [file veae093_supp.zip › FoIV1_FigureS6_revision.pptx]

## Slide 1
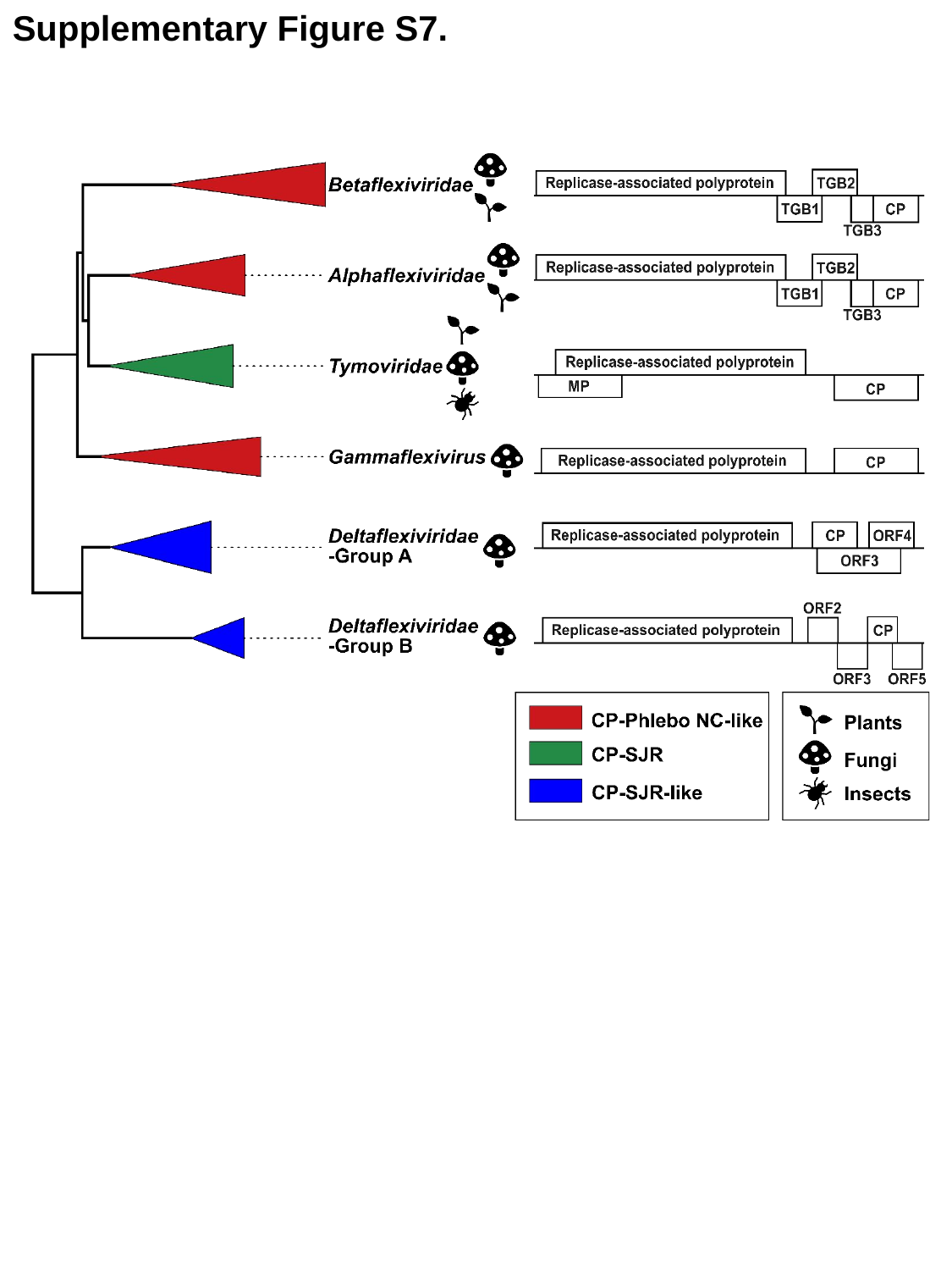

Supplementary Figure S7.

Supplement: veae093_Supp [file veae093_supp.zip › FoIV1_FigureS7_revision.pptx]
